# Aumolertinib: effective treatment for asymptomatic pulmonary giant cell carcinoma with *EGFR* L858R mutation - a case report

**DOI:** 10.3389/fonc.2023.1279045

**Published:** 2023-11-27

**Authors:** Wenxing Yang, Ze Yang, Kaiqiang Wang, Peiquan Zhu, Jiangtao Pu

**Affiliations:** Department of Thoracic Surgery, Affiliated Hospital of Southwest Medical University, Luzhou, China

**Keywords:** aumolertinib, PGCC, EGFR mutation, L858R, treatment

## Abstract

Aumolertinib, as a novel third-generation epidermal growth factor receptor tyrosine kinase inhibitor (*EGFR*-TKI), has been widely employed as a first-line treatment for advanced non-small cell lung cancer (NSCLC) patients with *EGFR* mutation. However, reports regarding the benefit of using aumolertinib as a monotherapy in pulmonary giant cell carcinoma are relatively scarce. In this report, we present a pulmonary giant cell carcinoma case harboring the *EGFR* Leu858Arg (L858R) mutation, with the patient at stage cT2bN3M1c IVB. Through the use of autolearning as a single agent, we effectively controlled the progression of pulmonary giant cell carcinoma, achieving a 6-month progression-free survival during the treatment course. Notably, the patient’s tumor not only ceased its growth but also continued to shrink, highlighting a significant therapeutic effect. This case reveals the effectiveness of aumolertinib as a monotherapy in controlling disease progression. The finding underscores the therapeutic advantage of aumolertinib in this particular subgroup of patients, offering a novel treatment option for pulmonary giant cell carcinoma.

## Introduction

Pulmonary giant cell carcinoma, a rare subtype within non-small cell lung cancer, accounts for only 0.11% of cases (1). Due to its complex pathological features, rapid clinical progression, poor prognosis, and frequent early metastasis, surgical treatment options are limited. Advanced non-small cell lung cancer treatment has undergone significant transformation in recent decades. EGFR-TKIs have become the first-line treatment for EGFR-positive patients. However, within the realm of pulmonary giant cell carcinoma, there remains a scarcity of reports on *EGFR* mutations and the solitary utilization of a drug like aumolertinib.

In this study, we present a pulmonary giant cell carcinoma case with the *EGFR* exon 21 L858R mutation. Treatment with aumolertinib led to significant reductions in tumor size and lymph node enlargement in the patient. Furthermore, we conducted a comprehensive literature review, exploring the advantages of aumolertinib in treating pulmonary giant cell carcinoma, while also investigating prevailing mechanisms of resistance and potential therapeutic strategies. This case not only contributes to the clinical understanding of pulmonary giant cell carcinoma but also provides valuable insights into the application of third-generation *EGFR*-TKIs for managing this uncommon subset of lung cancer.

## Case presentation

**Figure 1:**
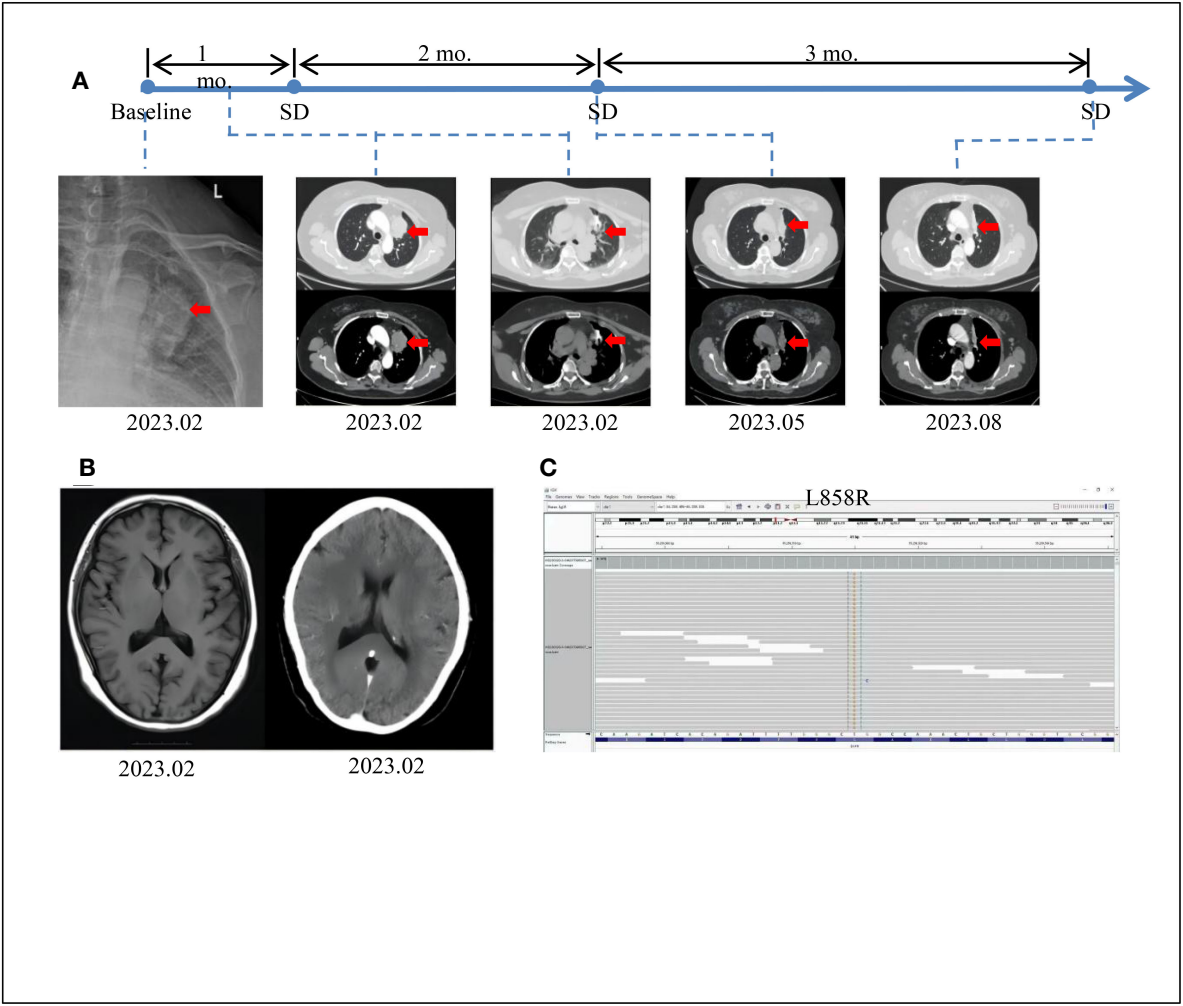
Tumor progression of the patient before and after treatment. **(A)** The timeline of therapies and tumor progression are indicated (Top). CT images revealed lesions in upper left lung. The tumor is indicated by red arrows. PFS, progression-free survival;SD, stable disease; PD, progressive disease; NGS, next-generation sequencing; mo., months. **(B)** Brain MRI scans revealed no metastasis at February 2023. **(C)** The EGFR exon21 c.T2573G p.L858R mutation was visualized by IGV software.

**Figure 2:**
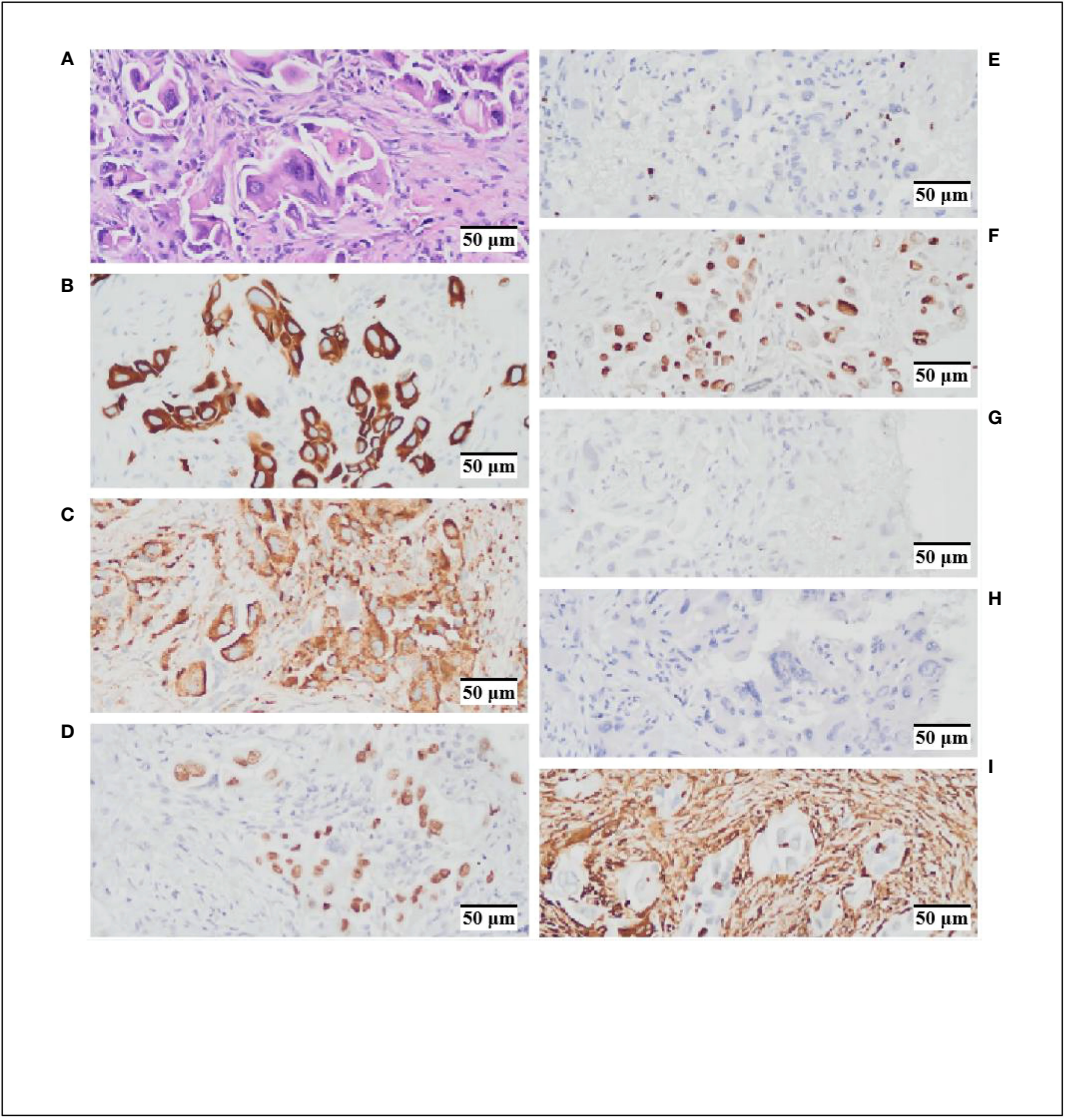
Hematoxylin and Eosin (HEx200) staining and immunohistochemistry (IHCx200) results. **(A)** HE staining indicates cytoplasmic acidophilia and nuclear basophilia, with evident intracellular cell proliferation features. **(B)** CK-7 (+). **(C)**. NaspinA (+). **(D)** TTF-1 (+). **(E)** Ki67 (+, 5%). **(F)** p53 (+, 10%). **(G)** CK5/6 (–) **(H)**. P40 (–) **(I)** VIM (–).

A 60-year-old female patient with no smoking history or family history of cancer presented in February 2023 with a 4-day history of left upper limb pain. Subsequent shoulder joint X-ray revealed a shadow in the left upper lobe of the lung (**Figure 1A**). Despite the absence of typical respiratory symptoms such as chest pain, chronic cough, fever, or shortness of breath, contrast-enhanced chest computed tomography (CT) disclosed a solid lesion within the left upper lobe measuring 5.0 x 3.7 cm, characterized by clear and lobulated margins. Furthermore, an enlarged right diaphragmatic lymph node was observed (**Figure 1A**). Fiberoptic bronchoscopy demonstrated patent left main bronchus, lobar bronchi, and segmental bronchi, with no evidence of new growth or mucosal erosion. Analysis of bronchoalveolar lavage fluid indicated a slight presence of neutrophils and macrophages, alongside a significant amount of epithelial cells. A bone scan revealed elevated basal metabolic activity, particularly in the right pubic ramus, suggesting potential metastasis. Subsequent CT-guided biopsy of the left upper lung revealed atypical giant cells within fibrous tissue (**Figure 1A**). The cranial magnetic resonance imaging (MRI) examination did not reveal any evidence of intracranial metastases (**Figure 1B**). Immunohistochemical analysis further confirmed its pulmonary origin, with positive markers including CK(+), CK7(+), NaspinA(+), TTF-1(+), Ki67(+, 5%), p53(+, 10%), CK5/6 (–), P40 (–), and VIM (–) (**Figures 2A–I**).To assess the possibility of distant metastasis, the patient underwent positron emission tomography-computed tomography (PET-CT), which indicated elevated glucose metabolism in the left upper lobe tumor. Additionally, lymph nodes near the aortic arch and left pulmonary hilum, as well as adjacent to the aorta and left portal vein, demonstrated potential signs of tumor metastasis. Notably, the possibility of bone metastasis was also considered, with the left humerus, bilateral pubic bones, and right ischium being potentially affected. Upon comprehensive evaluation of these clinical and investigative findings, the diagnosis of stage IVB pulmonary giant cell carcinoma (cT2bN3M1b according to the TNM staging system) was confirmed. By the recommendations of the National Comprehensive Cancer Center, chemotherapy intervention was initiated. The patient underwent one cycle of chemotherapy, including pemetrexed and carboplatin, which resulted in the emergence of nausea and vomiting symptoms. To alleviate these adverse effects, intravenous administration of granisetron hydrochloride was employed, leading to symptomatic relief and subsequent discharge.

Considering the patient’s strong aversion to chemotherapy-related adverse effects, a decision was made to explore more effective therapeutic options. Consequently, next-generation sequencing (NGS) was performed to assess genes related to lung cancer. The results revealed a clinically significant *EGFR* p.L858R-positive mutation (**Figure 1C**), accounting for an overall mutation rate of 5.68%. Following consideration of guidance from FNA, NMPA, NCCN, ASSO guidelines, and public databases, multiple treatment options were identified, including osimertinib, gefitinib, rociletinib, and aumolertinib. Considering the potent anti-resistance capabilities and lower side-effect profile of aumolertinib, the third-generation *EGFR*-TKI aumolertinib was chosen, with a daily dose of 110mg. Encouragingly, a follow-up chest CT after three months of aumolertinib treatment showed a significant reduction in the size of the tumor in the left upper lobe (4.4 x 1.9 cm) and the lymph node in the right cardiogenic angle (**Figure 1A**). Upon a follow-up examination in August, the tumor had decreased to 3.8x1.3cm, and the associated lymph nodes had also decreased in size (**Figure 1A**). In accordance with Response Evaluation Criteria in Solid Tumors version 1.1 (RECIST 1.1), the patient’s condition was classified as stable disease (SD). Given the patient’s favorable tolerability to aumolertinib and stable disease status, the decision was made to continue aumolertinib treatment. Presently, the patient remains on ongoing treatment with regular monitoring.

## Discussion

Pulmonary giant cell carcinoma, as a type of lung sarcomatoid carcinoma, is primarily characterized by a combination of multinucleated giant cells and neutrophils within an inflammatory milieu. Under high magnification, cells exhibit abundant cytoplasm, eosinophilia, smooth nuclear membranes, and small basophilic nucleoli. Additionally, conspicuous intracytoplasmic inclusions are observed, characterized by increased intracellular cellularity (**Figure 2A**). Immunohistochemical analysis reveals positivity for CK7(+), NaspinA(+), and TTF-1(+), implies a potential tendency toward glandular epithelial differentiation within the tumor. The positive expression of Ki67(+, 5%) and p53(+, 10%) suggests a certain degree of malignancy. In this case, we have identified a patient harboring a positive *EGFR* p.L858R mutation, coupled with the tumor’s propensity for glandular epithelial differentiation in the context of pulmonary giant cell carcinoma. This implies a potential benefit from *EGFR*-TKI treatment for the patient.

aumolertinib, a domestically developed third-generation TKI, gained domestic approval for marketing on March 18, 2020. Compared to the first-generation (gefitinib, erlotinib) and second-generation (afatinib) *EGFR*-TKIs, aumolertinib demonstrates enhanced stability and irreversible covalent binding with the ATP-binding domain of *EGFR*, efficiently inhibiting activating mutations (such as 19del and L858R) and resistance mutations (such as T790M), while displaying limited activity against wild-type (WT) *EGFR*. In contrast to osimertinib, aumolertinib introduces a cyclopropyl group to enhance stability (2, 3), allowing it to flexibly bind to the pocket of *EGFR*-T790M mutant protein, thereby increasing its affinity to T790M (2). Furthermore, it improves blood-brain barrier penetration in advanced NSCLC patients, suppressing brain and spinal cord metastases. In the Phase II clinical trial APOLLO, aumolertinib exhibits significant advantages owing to its unique anti-tumor properties. The primary endpoint, overall response rate (ORR), reaches 68.9%, and the secondary endpoint, median progression-free survival (PFS), extends to 12.4 months. Notably, patients with L858R mutations and exon 19 deletions achieve similar benefits in terms of PFS and overall survival (OS) (4). The design of aumolertinib, incorporating a cyclopropyl group, prevents robust inhibition of WT-*EGFR* metabolites’ production, markedly reducing other adverse effects such as diarrhea and rash induced by wild-type *EGFR* inhibition. The most common treatment-related adverse events (TRAEs) ≥10% include creatine phosphokinase (CPK) elevation (20.9%), rash (13.9%), aspartate aminotransferase (AST) elevation (12.3%), white blood cell (WBC) count reduction (12.3%), alanine aminotransferase (ALT) elevation (11.9%), and pruritus (10.7%); 15 patients (6.1%) experience prolonged QT interval, and there are no reports of interstitial lung disease (ILD) (36 cases) (4). Recently, a case of interstitial lung disease induced by aumolertinib was reported (5).

It has been observed that patients with PGCC carrying *EGFR* mutations lack significant and durable clinical responses to *EGFR* inhibitors (6, 7), which may be attributed to tumor resistance. Weng et al. reported two cases of PGCC patients receiving *EGFR*-TKI treatment. The first case demonstrated a favorable response to the treatment with tumor shrinkage, indicating potential benefits from gefitinib in the future. Conversely, the second patient, after receiving icotinib treatment, achieved a PFS of only 4.3 months, experiencing treatment failure with subsequent brain metastasis. *EGFR* mutation was detected in the tumor specimen obtained from the second surgical resection, with the persistent presence of *EGFR* exon 21 L858R gene mutation. The reasons for treatment failure are postulated to include insufficient brain penetration of icotinib to suppress tumor cell growth. Alternatively, the emergence of new *EGFR* mutations leading to treatment failure cannot be excluded. Unfortunately, the patient was not followed up after the second surgery (8). The most common mechanism underlying acquired resistance to first- and second-generation *EGFR*-TKIs is the T790M mutation, occurring in 50%-60% of cases (9). The third-generation aumolertinib can covalently bind to the T790M residue, suppressing the emergence of resistance, and is capable of attaining substantial concentrations in the brain and spinal cord, showcasing therapeutic potential for patients with bone and brain metastases.

Although aumolertinib demonstrates remarkable therapeutic efficacy, the issue of drug resistance should not be overlooked. Six major resistance mechanisms have been identified, including T790M deletion, persistent T790M presence, *EGFR* mutations (C797S, G724S, L718Q), activation of bypass pathways, transformation into small cell lung cancer (SCLC), and enhanced autophagy (10, 11). Regarding aumolertinib resistance mechanisms, they can be broadly categorized into two types: *EGFR*-dependent and *EGFR*-independent mechanisms. However, due to limitations in clinical research on aumolertinib, our understanding of its resistance mechanisms remains incomplete. Nevertheless, drawing insights from studies on osimertinib resistance mechanisms, we postulate that potential *EGFR*-dependent resistance mechanisms for aumolertinib may include the T790M mutation and *EGFR* point mutations. However, robust evidence for these mechanisms is currently lacking in the literature. Meanwhile, *EGFR*-independent resistance mechanisms are prevalent across various TKIs. Reports indicate that aumolertinib can sustainably activate downstream signaling pathways of *EGFR* through alternative pathways, including mTOR, ERK1/2, and STAT3, thereby reducing sensitivity to *EGFR*-TKIs (12). Furthermore, research suggests that certain patients exhibit EML4-ALK fusion mutations after aumolertinib treatment, implying ALK gene rearrangement as another potential mechanism for aumolertinib resistance (13). In conclusion, despite aumolertinib’s significant therapeutic potential, the diversity and complexity of its resistance mechanisms necessitate further in-depth research and understanding.

Combining aumolertinib with anti-angiogenesis therapy may enhance the effectiveness against pulmonary giant cell carcinoma. The rationale behind the combination of aumolertinib and anti-angiogenesis therapy is rooted in the latter’s ability to suppress tumor angiogenesis, thereby improving the delivery of *EGFR* TKIs through vascular normalization and enhancing their antitumor effects. As a *VEGFR*2 TKI, apatinib targets the intracellular domain of the receptor and disrupts signal transduction, consequently inhibiting tumor vascular growth. When used as a monotherapy, apatinib’s efficacy in advanced pulmonary giant cell carcinoma is not distinct. Li reported a case of palliative apatinib treatment for advanced pulmonary giant cell carcinoma that failed to restrain tumor progression. The patient received a nightly dose of 500mg aumolertinib, and after one month, the tumor had enlarged compared to before, along with an increase in bilateral lung metastases (14). Reliable research indicates that the combination therapy of third-generation *EGFR* TKIs such as osimertinib with immune checkpoint inhibitors (ICIs) or anti-angiogenesis agents only marginally improves median overall survival (mOS) and carries unpredictable safety concerns (15). Presently, there are no reports on the combination of aumolertinib with anti-angiogenesis therapy. Nevertheless, given the highly vascularized histology of pulmonary giant cell carcinoma, the combination of aumolertinib and anti-angiogenesis therapy holds potential within treatment strategies, necessitating further clinical validation.

The combination of aumolertinib with chemotherapy holds the potential to enhance the antitumor effects against PGCC. In the treatment landscape of advanced PGCC, chemotherapy remains an indispensable therapeutic approach. Notably, Fumihiro et al. reported a case of advanced PGCC where prolonged chemotherapy led to a complete remission lasting 15 months (16). Research has shown that the overexpression of ABC transporters contributes to increased drug efflux, a common mechanism of multidrug resistance (17). aumolertinib selectively inhibits the transport function of ABCB1 (MDR1/p-glycoprotein), suppressing drug efflux and restoring the sensitivity of ABCB1-overexpressing cancer cells to drug-induced apoptosis. Additionally, at submicromolar concentrations, aumolertinib effectively reverses ABCB1-mediated human multidrug resistance and maintains drug-induced apoptosis in ABCB1-overexpressing multidrug-resistant cancer cells (18), providing a theoretical basis for combining conventional cytotoxic anticancer drugs with aumolertinib. In a clinical retrospective study, a cohort of 50 patients received single-agent aumolertinib as first-line treatment, while 15 patients underwent combination therapy (pemetrexed administered one week before aumolertinib). The combination therapy group exhibited significantly higher objective response rates (ORR) and disease control rates (DCR) of 93.3% and 100%, respectively, compared to the single-agent aumolertinib group with rates of 64% and 92%. Among the combination therapy recipients, 5 patients with *EGFR* mutations observed notable tumor reduction after 2-3 treatment cycles. Among these, 4 patients transitioned from clinical stage III/IV to postoperative pathological stage I, and 1 patient achieved a complete pathological response from clinical stage IIIB to postoperative pathological stage T0N0M0 (19). In our case, we adopted a strategy of administering pemetrexed before initiating aumolertinib treatment. In the absence of a definitive diagnosis of the lung tumor type, we employed a treatment regimen comprising paclitaxel and carboplatin. After confirming the *EGFR* mutation type two weeks later, the patient continued aumolertinib treatment, without the periodic addition of pemetrexed and aumolertinib. This suggests that periodic use of pemetrexed and aumolertinib could potentially yield greater benefits.

In summary, this is the inaugural report of a rare case of EGFR L858R mutation-positive stage IV pulmonary giant cell carcinoma that has exhibited marked benefits from exclusive utilization of Aumolertinib. As of the present moment, the patient has undergone continuous Aumolertinib treatment for six consecutive months, resulting in substantial tumor regression and an absence of any adverse reactions. These outcomes underscore the remarkable therapeutic potential of Aumolertinib in the treatment of pulmonary giant cell carcinoma. Given the intricate tumorigenic mechanisms, long-term monotherapy with Aumolertinib often precipitates issues of drug resistance. To address this concern, a limited analysis of Aumolertinib resistance mechanisms was undertaken. The scrutiny of relevant literature revealed that the combination of Aumolertinib with concurrent chemotherapy and anti-angiogenic therapies may offer substantial therapeutic advantages. Consequently, we emphasize the distinctive attribute of Aumolertinib as a standalone therapeutic modality for pulmonary giant cell carcinoma, along with its potential when integrated into combination therapies, as pivotal avenues for extending the survival of patients with EGFR-positive pulmonary giant cell carcinoma.

## Data availability statement

The original contributions presented in the study are included in the article/supplementary material, further inquiries can be directed to the corresponding author/s.

## Ethics statement

The studies involving humans were approved by Affiliated Hospital of Southwest Medical University. The studies were conducted in accordance with the local legislation and institutional requirements. The human samples used in this study were acquired from Inpatient routine pathological biopsy and immunohistochemistry. Written informed consent for participation was not required from the participants or the participants’ legal guardians/next of kin in accordance with the national legislation and institutional requirements. Written informed consent was obtained from the individual(s) for the publication of any potentially identifiable images or data included in this article.

## Author contributions

WY: Data curation, Resources, Writing – original draft, Writing – review & editing. ZY: Data curation, Resources, Software, Writing – original draft, Writing – review & editing. KW: Resources, Writing – review & editing. PZ: Writing – review & editing. JP: Funding acquisition, Writing – review & editing.
